# Use of post-unit hospital deaths of ICU survivors as a quality indicator

**DOI:** 10.1186/2197-425X-3-S1-A364

**Published:** 2015-10-01

**Authors:** M Argent, A Clarke

**Affiliations:** Department of Anaesthetics and Intensive Care, Royal Gwent Hospital, Aneurin Bevan University Health Board, Newport, United Kingdom

## Introduction

ICNARC publish a number of quality indicators for the South Wales critical care network. One of these is the number of post-ICU hospital deaths for ICU survivors. In the April 2012-March 2013 report our unit had the highest number of the patients in South Wales, being above network and case-mix programme (CMP) average.

## Objectives

This triggered us to review the causes of death in patients after ICU discharge to learn if there were modifiable factors and to ensure optimal allocation of critical care resources.

## Methods

We conducted a retrospective case review for all patients who died after ICU discharge but within hospital in the year 1 April 2012 to 31 March 2013.

## Results

There were 796 critical care admissions in the specified period. Of these, 60 (7.5%) patients died in-hospital after being discharged from ICU. Nine (15%) patients were discharged with treatment limitations in place and suffered further deterioration after ICU.

Another nine (15%) patients were discharged with a new diagnosis of end-stage non-malignant disease (predominantly liver or renal failure). End stage malignancy was diagnosed in seven (12%) patients during their ICU stay and re-admission was not felt appropriate. A total of ten (17%) patients suffered unexpected complications after ICU discharge, but readmission was deemed inappropriate in seven (70%) of these patients. Seven (12%) patients were coded incorrectly, and had actually been discharged from hospital alive.Two (3%) patients were re-admitted and died on the unit and two (3%) were transferred to other hospitals.

Notes were not available for review for 14 (23%) patients.

## Conclusions

ICNARC return the rate of post-ICU in-hospital deaths as a quality marker to identify whether use of critical resources could have been avoided. Our review reveals a number of factors regarding use of this quality indicator. Firstly, many (16, 27%) patients who died in hospital after surviving ICU had end-stage disease diagnosed during their ICU stay. These figures are consistent with a previous prospective study [2]. Prognostication is a complex area, and whilst using robust scoring systems may prevent further admissions of this nature, it is likely that this scenario cannot be entirely removed. This review has been useful to identify the factors which affect this quality indicator, and identify that our data capture and processing could be improved. We did not identify any cases in which critical care admission could have been avoided, given the information that was available to the clinician at the time of admission.

## Acknowledgment

We would like to thank Linda Garland ICNARC Data Clerk at the Royal Gwent Hospital for her assistance.
